# Supplementary material for: KLF9 Aggravates Streptozotocin-Induced Diabetic Cardiomyopathy by Inhibiting PPARγ/NRF2 Signalling
Source: Cells. 2022 Oct 27;11(21):3393. doi: 10.3390/cells11213393 (PMC9656075; doi:10.3390/cells11213393)
Supplement: Supplementary file 1 [file cells-11-03393-s001.zip › cells-1923415-supplementary.pdf]

Supplementary Materials

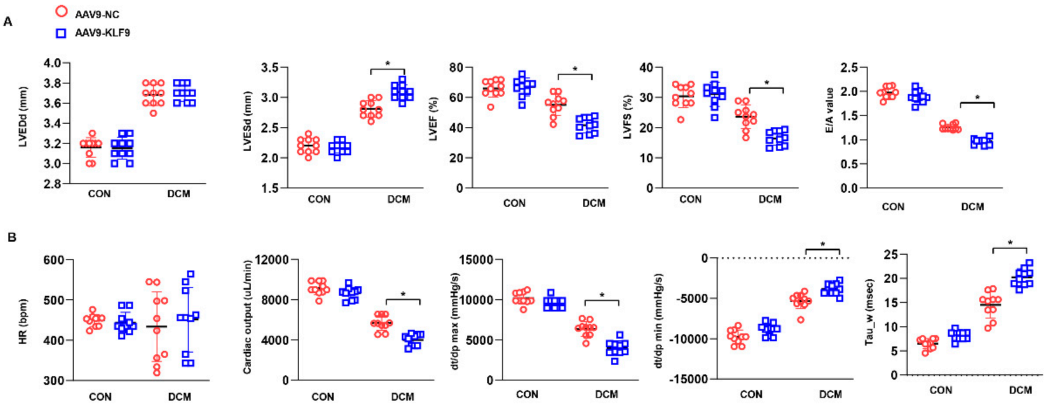

Figure S1. KLF9 overexpression deteriorated cardiac dysfunction in DCM. (**A**) Echocardiographic data in DCM mice (n=10). (**B**) Pressure-volume loop data in DCM mice (n=10). *P<0.05.

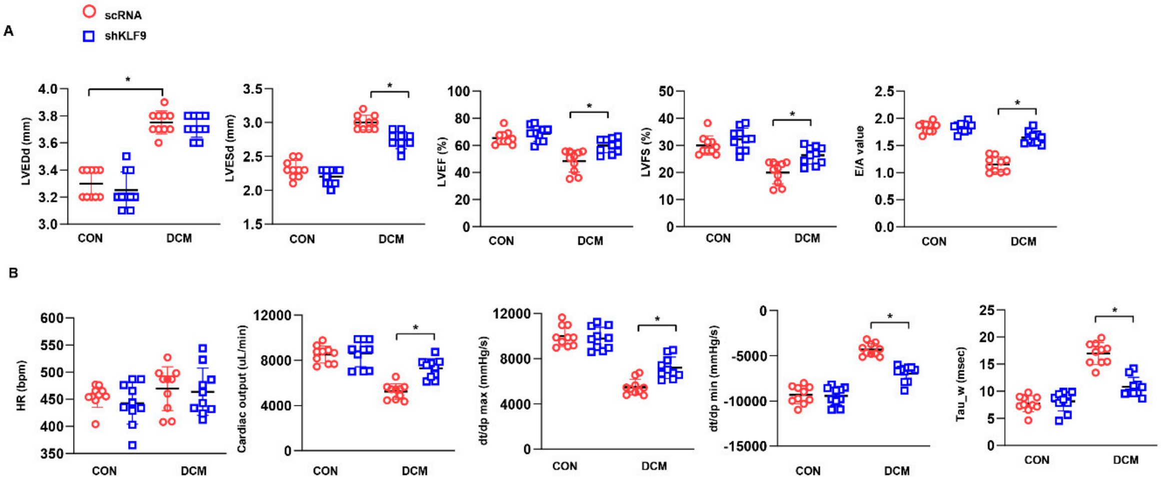

Figure S2. KLF9 knockdown ameliorated cardiac dysfunction in DCM (**A**) Echocardiographic data in DCM mice (*n*=10). (**B**) Pressure-volume loop data in DCM mice (n=10). *$P < 0.05$.

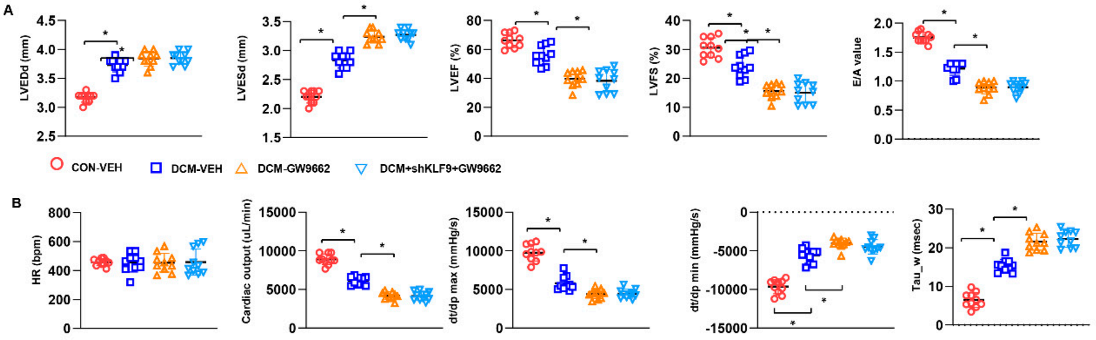

Figure S3. Cardiac dysfunction persists in PPARγ inhibition mice with KLF9 knockdown. (A) Echocardiographic data in DCM mice ($n = 10$). (**B**) Pressure-volume loop data in DCM mice ($n = 10$). *$P < 0.05$.
